# The role of gut microbiome and its metabolites in pancreatitis

**DOI:** 10.1128/msystems.00665-24

**Published:** 2024-08-30

**Authors:** Letian Pan, Nuoming Yin, Mingyu Duan, Qixiang Mei, Yue Zeng

**Affiliations:** 1 Shanghai Key Laboratory of Pancreatic Disease, Shanghai JiaoTong University School of Medicine, Shanghai, China; 2 Department of Gastroenterology, Shanghai General Hospital, Shanghai JiaoTong University School of Medicine, Shanghai, China; 3 Shanghai JiaoTong University School of Medicine, Shanghai, China; Chinese Academy of Sciences, Beijing, China

**Keywords:** gut microbiome, metabolites, pancreatitis, microbiome-based treatment, diet

## Abstract

Gut microbiome plays a vital role in the intestinal ecosystem and has close association with metabolites. Due to the development of metabolomics and microbiomics, recent studies have observed that alteration of either the gut microbiome or metabolites may have effects on the progression of pancreatitis. Several new treatments based on the gut microbiome or metabolites have been studied extensively in recent years. Gut microbes, such as *Bifidobacterium*, *Akkermansia*, and *Lactobacillus*, and metabolites, such as short-chain fatty acids, bile acids, vitamin, hydrogen sulfide, and alcohol, have different effects on pancreatitis. Some preliminary studies about new intervention measures were based on the gut microbiome and metabolites such as diet, prebiotic, herbal medicine, and fecal microbiota transplantation. This review aims to summarize the recent advances about the gut microbiome, metabolites, and pancreatitis in order to determine the potential beneficial role of the gut microbiome and metabolites in pancreatitis.

## INTRODUCTION

The pancreas is an important organ in the human body composed of acinar cells, duct cells, and islet cells (α cells, β cells, δ cells, and PP cells). The pancreas functionally consists of two parts: the exocrine pancreas containing acinar cells and ductal cells, and the endocrine pancreas including islets cells (1). Pancreatitis, triggered by injury to acinar cells and activation of trypsinogen, features inflammation, exudation, hemorrhage, necrosis, and fibrosis in the pancreas (2). Pancreatitis may develop into severe acute pancreatitis (SAP) that results in local complications and organ dysfunction (3). Uncontrolled pancreatitis, triggering consistent acinar cell and islet cell injury, may transform into pancreatic cancer or diabetes mellitus (4, 5).

Gut microbiome plays a vital role in the gut ecosystem, protecting the gut barrier and regulating host metabolism (6). Previous studies have discovered that the gut microbiome can make effects on the progression of pancreatitis (7). It is widely recognized that metabolites are strongly affected by the microbiome (8, 9). In recent years, metabolite–disease relationships are being brought into sharp focus profiting from the application of bioinformatics analysis. Nowadays, metabolites have been found to cause inflammation in obesity, Alzheimer’s disease, non-alcoholic fatty liver disease (NAFLD), inflammatory bowel disease (IBD), etc. Such relationships have also been demonstrated in pancreatic diseases (10). Therefore, there is a strong possibility that metabolites, which are closely linked with the microbiome, play an essential role in pancreatitis. In the past decades, studies on the gut microbiome, metabolites, and pancreatitis were limited. However, due to the booming development of metabolomics and microbiome in the field of pancreatitis, more and more studies have found links between the gut microbiome, metabolites, and pancreatitis in recent years. In this review, we will summarize how microbiome affects pancreatitis through the regulation of metabolites. Furthermore, we will discuss the mutual effects of metabolites and microbiome in pancreatitis. Finally, we will introduce the intervention measures that affect the gut microbiome and metabolites in pancreatitis.

## GUT MICROBIOME AND ITS METABOLITES IN PANCREATITIS

**Fig 1:**
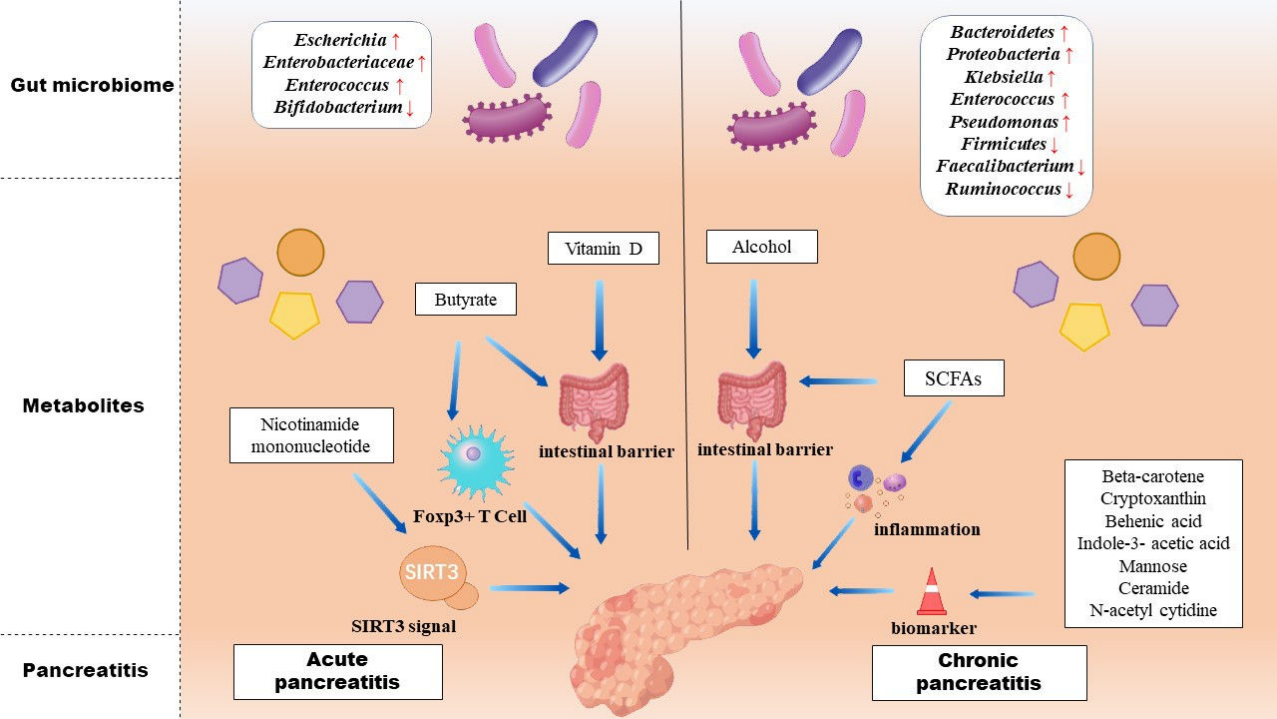
Relationship between the gut microbiome, metabolites, and pancreatitis. Changes in intestinal flora occur during the development of pancreatitis, resulting in changes in metabolites, which, in turn, have multiple effects on pancreatitis.

Pancreatitis is mainly composed of acute pancreatitis (AP), chronic pancreatitis (CP), and other associated pancreatic diseases (11). In recent years, researchers have used metabolomics and microbiomics to examine the vital role of metabolites and the gut microbiome in the human body. The relationship between pancreatitis, metabolites, and microbiome has been demonstrated in recent studies (Fig. 1).

### Acute pancreatitis

Acute pancreatitis is a non-infectious inflammation of the pancreas with acute damage. Approximately 20% of AP converts into SAP because of intestinal barrier damage, microbiome translocation, and microbiota dysbiosis (12). Studies have shown that the structure of the gut microbiota was different between AP patients and healthy people. Opportunistic pathogenic bacteria such as *Escherichia–Shigella* increased, and beneficial bacteria such as *Bifidobacterium* decreased in the progression of AP development (13). In the experiment of Tan et al. (14), there is an increase in pathogenic bacteria such as *Enterobacteriaceae* and *Enterococcus*, and a decrease in beneficial bacteria such as *Bifidobacterium* in AP patients compared to healthy individuals. Recently, it has been suggested that certain kinds of metabolites altered by the alteration of the microbiota act as protective factors in AP progression, such as butyrate, vitamin D, and nicotinamide mononucleotide (NMN) (15–17).

Butyrate, the short-chain fatty acids (SCFAs) with four carbon atoms, makes protective effects on AP from multiple aspects. In the SAP study related to Western diet, the supplement of butyrate significantly decreased the mortality (15). Several studies confirmed that butyrate reduced inflammatory cell infiltration and inflammatory factor expression in AP (18–20). In this progression, nuclear factor κB (NF-κB) and histone deacetylase (HDAC) play an important role (21). Butyrate could significantly inhibit the mutual actions between HDAC and AP1 and STAT1, and then suppress the activation of NLRP3 inflammasomes (20). In addition, recent studies focused on the effects of butyrate on immune response. For instance, Foxp3+ regulatory T cells can be raised by butyrate during SAP. Besides the protective effects on the pancreas, butyrate could alleviate intestinal barrier damage in AP (18). Butyrate attenuates SAP through inflammation regulation, immune response regulation, and intestinal barrier protection, which makes it a promising research target.

Deficiency of vitamin D may predict SAP (17). Vitamin D is known to be associated with calcium metabolism. Hypercalcemia secondary to vitamin D deficiency could lead to pancreatic duct obstruction and pancreatic vasculitis, which increased the incidence of AP (22). Moreover, vitamin D has been found to play a vital role in the regulation of inflammation via vitamin D receptor (VDR) (23). What’s more, vitamin D can protect the intestinal barrier that is damaged during SAP (23). However, excess vitamin D has also been reported to aggravate AP (24). The toxicity of the serum vitamin D level threshold ranges from 50 to 150 ng/mL in different studies (22). In summary, vitamin D, as a common nutrient in daily life, is a promising research direction, especially for anti-inflammatory studies through VDR. The detailed mechanism of vitamin D’s regulation of inflammation in AP remains elusive.

Nicotinamide mononucleotide related to *Prevotellaceae* alleviates acute pancreatitis by activating pancreatic SIRT3 signal (16). Other metabolites, such as acetate, bile acids (BAs), polyamines, and so forth, are also reported to affect the progression of AP.

### Chronic pancreatitis

Once acute pancreatitis is not treated in time, it may convert to CP (2). CP causes continuous pain and exocrine and endocrine pancreatic deficiency, resulting in lower quality of life (25). Furthermore, CP raises the risk of pancreatic cancer and diabetes mellitus (26). Previous studies have confirmed significant changes in the microbiota of CP (27). For example, in CP mice, there are more *Bacteroidetes* and *Proteobacteria* and less *Firmicutes* compared with control mice (27).

Different metabolites produced by the microbiome may have different effects on CP. Endotoxin increased from controls to CP non-diabetics to CP diabetics, whereas *Faecalibacterium prausnitzii* and *Ruminococcus bromii* declined (28). Part of alcohol-induced organ injury is related to microbiota dysbiosis. The small intestinal bacterial overgrowth caused by alcohol abuse may contribute to gut leakiness (29). Ciocan et al. (30) found that the abundances of several potential pathogenic microbes, such as *Klebsiella*, *Enterococcus*, and *Pseudomonas*, were higher in chronic alcoholic pancreatitis. In addition, other researchers found that soy bread diet could protect intestinal barrier and reduce inflammation in CP. This may be due to the gut microbiota, which fermented indigestible starches and complex sugars into SCFAs (31).

Besides, a prediction model based on blood plasma and serum metabolomics has been established to distinguish CP from non-pancreas diseases (32). Beta-carotene, cryptoxanthin, behenic acid, indole-3-acetic acid, ceramide, mannose, and N-acetyl cytidine were used as biomarkers in CP patients.

### Autoimmune pancreatitis

Except for AP and CP, autoimmune pancreatitis (AIP) is also related to microbiome.

AIP is a type of special pancreatitis presenting with increased immunoglobulin levels and duct destruction (33). AIP is divided into two subtypes: type 1, common and associated with IgG4, and type 2, rare and not associated with IgG4 (34). AIP shows chronic fibro-inflammatory like CP, but it is reversible (35). Thus, no consensus has been reached on defining AIP as a subtype of CP yet (36).

Many bacteria are confirmed related to AIP. Kamata et al. (37) found that *Klebsiella pneumoniae* increased the severity of AIP by activating plasmacyte-like dendritic cells, which produce IFN-α and IL-33. The relationship between *Helicobacter pylori* infection and autoimmune pancreatitis has been documented (38). Besides, Hamada et al. (39) examined the gut microbiota of AIP and found that some bacterial species, such as *Streptococcus australis* and *Streptococcus gordonii*, were less abundant in AIP.

According to previous research, microbiota mostly affect the progression of AIP via immune cells. However, whether microbial metabolites have effects on AIP or not remains to be studied.

## MICROBIOME-ASSOCIATED METABOLITES IN PANCREATITIS

Metabolites are products and intermediates of cellular metabolism (40). Metabolites serve a multitude of functions in the progression of diseases, including energy conversion, microbiota regulation, signaling, and so on (40). Besides, metabolites are recognized as a critical bridge between the gut microbiome and the host (41). Commensal or pathogenic microbiota produce diverse kinds of metabolites to have effects on the host. Furthermore, microbiota can transfer the metabolism of host products to a variety of primary and secondary metabolites.

Microbiome mainly affects metabolites via biosynthesis, transformation, receptor regulation, and immune signaling (42–44). The effects of metabolites are observed in diseases closely related to the gut microbiome such as IBD (45), NAFLD (46), obesity (47), and hepatic cancer (48). The relationship between microbiota and metabolites is usually found in these diseases. In pancreatitis, microbiome and microbial metabolite have also been documented as important factors (49). We found that SCFAs, BAs, vitamins, and hydrogen sulfide (H_2_S) were relatively more strongly associated with different pancreatitis. Some related microbes are listed in Table 1.

**TABLE 1:** Relationship between the gut microbiome and metabolites^*a*^

Metabolites	Gut microbiome	Physiological function	References
Acetate	*Parabacteroide*↑ *Blautia*↑	Alleviate AP Recover the microbiota dysbiosis caused by alcohol	(50) (13) (51) (52)
Propionate	*Akkermansia*↑ *Bacteroidetes*↑ *Firmicutes*↑	A precursor of glucose synthesis in the liver Inhibit apoptosis Modulate Ca^2+^ channel Inhibit the proliferation of tumors Enhance glucose-stimulated insulin Release and keep β-cell mass	(53) (54) (55) (56) (57)
Butyrate	*Clostridium*↑ *Butyrivibrio*↑ *Lachnoclostridium*↑ *Eubacterium*↑ *Faecalibacterium*↑ *Eubacterium*↑ *Odoribacter*↑ *Splanchnicus*↑	Alleviate colonic injury associated with AP Regulate the inflammatory response by inhibiting the NLRP3 inflammasome pathway	(58) (20) (59) (60) (61)
Bile acids	*Clostridium scindens*↑ *Muribaculum*↑ *Clostridium*↑ *Muribaculum*↑ *Bacteroides*↑ *Bifidobacterium*↑ *Actalibacter*↑ *Akkermansia*↑	Induce cell death by impairing cellular Ca^2+^ signaling Reduce pancreatic and intestinal injury Overexpressed MUC4 expression Inhibit the action of pancreatic cancer (PC) cells	(62) (63) (64) (65) (66) (67)
Vitamin A		Regulate pancreatic development β-Cell function Pancreatic innate immune responses Pancreatic stellate cell phenotypes Enhance gut microbiota diversity Enhance glucose-stimulated insulin secretion Enhance β cell mass	(68) (69)
Vitamin B_12_	*Bifidobacterium*↑ *Actinobacteria*↑ *Fusobacterium*↑ *Lactobacillus*↑	Suppress oxidative stress Improve mitochondria dysfunction Alleviate AP	(70) (71) (72) (73)
Vitamin D	*Lactobacillus*↑	Protect gut barrier Anti-inflammatory Affect innate immunology Prevent pathological dedifferentiation of pancreatic β cells Prevent SAP	(23) (74) (75) (76) (17)
Hydrogen sulfide	*Archaeoglobus*↑ *Desulfotomaculum*↑ *Thermodesulfovibrio*↑ *Deltaproteobacteria*↑	Act as gaseous signaling molecule Pro-inflammatory Inhibit gut motility Promote the inflammatory response caused by SAP Exacerbate AP and CP Activate autophagy excessively	(77) (78) (79) (80) (81) (82)
Ethanol	*Bacteroides*↑ *Escherichia*↑ *Bifidobacterium*↑ *Clostridium*↑	Aggravate CP Accumulate fatty acid ethyl esters Induce autophagy Exacerbate fibroinflammatory in CP Trigger microbiota dysbiosis	(83) (84) (85) (29)
Indoles	*Escherichia coli*↑ *Vibrio cholerae*↑ *Peptostreptococcus*↑ *Lactobacillus reuteri*↑	Protect intestinal barrier integrity Regulate immune cell activity Prevent postoperative pancreatitis Activate AhR, which promotes tumor growth via inhibiting CD8+ T cells	(86) (87) (88) (89) (90)
Lactate	*Bifidobacterium*↑	Reduce pancreatic injury in AP Suppress inflammation	(91) (92)

^*a*^ “↑” means that the gut microbiome increases the metabolite or promotes its function, and “↓” means that the gut microbiome decreases the metabolite or suppresses its function.

### Short-chain fatty acids

SCFAs refer to saturated fatty acids that have a carbon chain of no more than six atoms. They affect the host as a regulator of the gut metabolism, gut endocrine, proliferation, and differentiation, and the guard of the gut barrier (53). SCFAs are derived from dietary fibers. The digestion of dietary fibers needs some certain enzymes, which are only derived from gut microbes. These microbes degrade dietary fibers into SCFAs, gas, and organic acids (53). Acetate, propionate, and butyrate are the main components of SCFAs in the human body.

Acetate plays a vital role in pancreatitis and related pancreatic diseases. Acetate is a product of most gut microbes (53). *Parabacteroide* is found to produce acetate to regulate neutrophil infiltration to alleviate AP (50). *Blautia*, a widely known acetate producer (13), is found to alleviate AP via acetate (51). In cachexia patients of pancreatic cancer, the abundance of *Proteobacteria* and *Veillonella* increased, whereas acetate decreased (93). Besides, Martino et al. found that acetate could recover the microbiota dysbiosis caused by alcohol in the gut (52), which implied that acetate may be able to alleviate chronic alcohol pancreatitis.

Propionate has been found to have several important functions in the human body such as being a precursor of glucose synthesis in the liver, inhibiting apoptosis, inhibiting cell proliferation, and modulating Ca^2+^ channel via GPR42 (53, 54). Propionate is produced by succinate pathway or propanediol pathway (53). *Bacteroidetes* and *Firmicutes* produce propionate in the former way, whereas some bacteria, such as *Akkermansia muciniphila*, do so in the latter way (55). Propionate is found to inhibit the proliferation of tumors (56). Besides, propionate can improve β-cell function because it can enhance glucose-stimulated insulin release and keep β-cell mass via inhibiting apoptosis (57). More studies on the association between propionate and pancreatitis remain to be conducted.

Butyrate is a mediator of the immune response and microbiota regulation in the human body (94). It has been proven to be produced by *Clostridium*, *Butyrivibrio*, *Lachnoclostridium*, *Eubacterium*, and *Faecalibacterium* (58). Butyrate and its producers have been found to have a close link to AP. Butyrate was reported to alleviate gut injury associated with AP and inflammatory response in the pancreas via suppressing the NLRP3 inflammasome pathway (20). *Clostridium butyricum*, which produces butyrate, regulates the microbiome and alleviates the inflammation in AP through NLRP3 and MMP9 pathways (59, 60).

### Bile acids

Bile acids consist of primary bile acids and secondary bile acids. Microbes are essential in the procession of BA transformation (95). In bile acid conversion, *Clostridium scindens* takes part in 7α-dehydroxylation, *Muribaculum* and *Bacteroides* take part in dihydroxylation, and six other microbes (*Clostridium*, *Muribaculum*, *Bacteroides*, *Bifidobacterium*, *Actalibacter*, and *Akkermansia*) take part in oxidation (62). Microbes remove glycine or taurine conjugates to prevent bile acids from being reabsorbed from the small intestine (96). This procession is finished by the microbes with bile salt hydrolase (BSH). Macrogenomics research shows that BSH exists in all major bacteria and archaea in the human intestinal tract, such as *Lactobacillus*, *Bifidobacterium*, *Clostridium*, and *Bacteroides* (63). What’s more, intestinal bacterial overgrowth leads to abnormal bile acid decomposition and nutrient absorption (97). In addition to the classic function in digestion, BAs play as an inflammation regulator (98).

BAs are found to trigger the injury of pancreatic acinar cells through cellular Ca^2+^ signaling in AP (64). Receptors interacting with BAs in pancreatic acinar cells include Gpbar1, FXR, RyR, IP3Rs, and TRPV1 (98). The enhancement of Ca^2+^ ions induces mitochondrial membrane depolarization and activation of phosphatidylinositol 3-kinase (PI3K). BAs also damage pancreatic stella cells during pancreatitis. Sodium-dependent transport protein may play an important role in the effects of BAs on pancreatic stella cells. Studies on the effects of BAs on pancreatic stella cells remain elusive. In intervention research, BA supplementation could reduce pancreatic and gut damage. It is reported that *Lactobacilli* are the key bacteria here (65).

### Vitamins

Vitamins are organic compounds that are essential for body growth and optimal nutrition, but they cannot be synthesized by the human body (99). Vitamin A, vitamin B12, and vitamin D have been reported to be more important than other vitamins in pancreatitis (72). Microbiome plays an important role in vitamin synthetization.

Vitamin A is ingested in the form of either retinyl esters or carotenoids, and transformed to active compounds such as all-trans-retinoic acid (100). Vitamin A is observed to affect organ development, β-cell function, innate immune responses, and stellate cell phenotypes in the pancreas via several mechanisms (68). Zhou et al.’s experiment found that mice fed with a vitamin A-deficient diet had lower gut microbiota diversity, lower glucose-stimulated insulin secretion, and lower β cell mass than those fed with a vitamin A-sufficient diet, and vitamin A could make up for the backwardness (69). Moreover, a higher gut microbiota diversity is related to a higher glucose-stimulated insulin secretion and β cell mass in this experiment. Thus, microbes are involved in vitamin A’s effects on blood glucose.

Vitamin B12, which is also called cobalamin, can only be synthesized by microbes (101). It is synthesized by certain bacteria and archaeon (102). *Bifidobacterium*, *Actinobacteria*, *Fusobacterium*, and *Lactobacillus* are found to be involved in the production of vitamin B12 (70, 71). Vitamin B12 can alleviate acute pancreatitis through the inhibition of oxidative stress and the enhancement of mitochondria dysfunction (72). In islet cell dysfunction mediated by nicotine, vitamin B12 alleviates the mitochondrial dysfunction caused by nicotine to alleviate the islet cell dysfunction (73).

Vitamin D, mainly produced by the skin, is a fat-soluble vitamin. Vitamin D makes biological effects through VDRs. In addition to calcium metabolism and bone homeostasis, vitamin D has been found to affect innate immunology (74). There is a positive relationship between VDR and anti-inflammatory (23). Deficiency of vitamin D is found to aggravate inflammation via NOD2/CARD15-defensin beta2 innate immune pathway (75). Microbes are found to affect the abundance of VDR. Pathogenic bacteria may reduce the amount of VDR (103). *Lactobacillus* has been observed to increase the amount of VDR (104). Meanwhile, a toll-like receptor is found to be a trigger of a vitamin D-mediated antimicrobial response (105). Vitamin D deficiency may predict SAP (17). This may be because vitamin D can protect the gut barrier, which plays an important role during AP (23). Furthermore, a VDR-targeted treatment to prevent pathological dedifferentiation of β cells under hyperglycemic stress was documented (76).

### Hydrogen sulfide

H_2_S, once thought to be a toxic gas, is recently found as an endogenous regulatory factor with extensive physiological functions (106). H_2_S is mainly produced by sulfuric acid-reducing bacteria such as *Archaeoglobus*, *Desulfotomaculum*, *Thermodesulfovibrio*, and *Deltaproteobacteria*. Some sulfuric acid-reducing bacteria may produce SCFAs at the same time (107). In human colon and other tissues, H_2_S is synthesized from cysteine via cysteine γ-lyase (CSE) and cystathionine β-synthase (CBS). H_2_S biogenesis by cysteine metabolism is a critical biochemical output of the human gut microbiota (108). Seven cysteine-degrading enzymes were found in the genomes of *Bacteroidesovatus* and *Enterococcus faecalis,* which take part in inflammation (109).

H_2_S has been reported to regulate inflammation as a gas signaling molecule (78). It may be closely linked with pancreatitis. In acute pancreatitis, H_2_S inhibits gut motility and promotes inflammatory response in SAP (79). PI3K/Akt/Sp1 pathway is associated with the enzymes producing H_2_S such as CSE and CBS. The inhibition of the PI3K/Akt/Sp1 pathway could reduce the production of CSE and CBS. The pro-inflammatory effects of H_2_S may be induced through SP-NK-1R pathway (80). Meanwhile, the supplement of DL-propargylglycine, an inhibitor of CSE, significantly alleviated the inflammation in AP and reduced the substance P in the SP-NK-1R pathway. Furthermore, H_2_S could exacerbate AP by activation of autophagy excessively by AMPK/mTOR pathway (81). Except for AP, the elevated H_2_S level may be significantly associated with CP, but more information remains to be found (82).

### Other metabolites

Other gut microbial metabolites, such as indoles, lactate, ketone, and ethanol, were found to be associated with certain kinds of pancreatitis. Their effects potentially work in different ways—exacerbating or alleviating, directly or indirectly.

Indoles are important gut bacterial metabolites mainly derived from aromatic amino acids. They can make impacts on the gut barrier and immune cell activity via aryl hydrocarbon receptor (AhR) and pregnane X receptor (PXR) (87, 88). Several species of bacteria have been shown by numerous studies to be capable of converting tryptophan to indole such as *Escherichia coli*, *Vibrio cholerae*, and *Peptostreptococcus* (86). *Lactobacillus reuteri* is a major producer of tryptophan and indoles, and can inhibit pro-inflammatory activity via stimulating AhR (110).

Lactate is derived from intestinal bacterial fermentation of carbohydrate. Lactate alleviates pancreatic damage in AP by inhibiting toll-like receptor and inflammasome-mediated inflammation (92). *Bifidobacterium* spp. are found to produce lactate to alleviate AP via inhibiting inflammatory responses (91).

Alcohol (ethanol) abuse is a dangerous factor of CP (111). In addition to ethanol deriving from alcohol, ethanol can be synthesized by gut bacteria such as *Bacteroides fragilis*, *Escherichia*, *Bifidobacterium adolescentis*, and *Clostridium thermocellum* (83). Ethanol aggravates CP through accumulation of fatty acid ethyl esters (FAEEs), which are the nonoxidative metabolites of ethanol, through induction of autophagy, and through interaction with other diseases (84). Impaired autophagy can increase susceptibility to endotoxin-induced chronic pancreatitis (112). Chronic excessive ethanol and FAEE exposure exacerbated fibro-inflammatory in CP (85). Besides, ethanol exacerbates CP via microbiota dysbiosis. Gut bacterial overgrowth caused by ethanol abuse relates to gut leakiness (29). Relative abundance of several potential pathogenic microbes, such as *Klebsiella*, *Enterococcus*, and *Pseudomonas*, is higher in chronic alcoholic pancreatitis (30).

## INTERVENTION MEASURES BASED ON GUT MICROBIOTA AND ITS METABOLITES

### Diet

**Fig 2:**
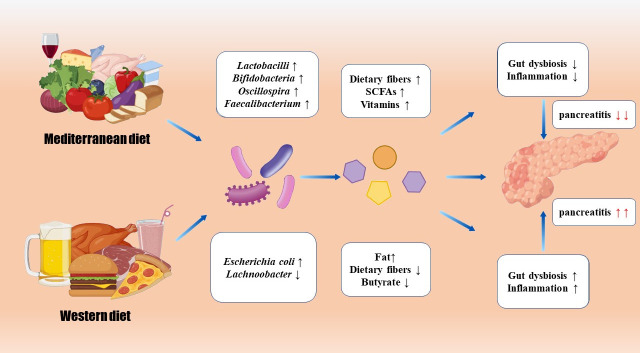
Comparison between Mediterranean diet and Western diet in pancreatitis. Mediterranean diet and Western diet have different effects on the gut microbiome and metabolites, and each respectively either alleviates or aggravates pancreatitis.

All ingested food undergoes metabolic transformations and is converted into metabolites and other compounds within the gastrointestinal tract. In this progression, microbiota may take part in metabolite production. Thus, different diets may impact diseases by altering the microbiota and metabolites (Fig. 2).

The Western diet is well known for lacking dietary fibers and being high in fat. This diet type is found to change the structure of microbiota and causes metabolic inflammation so that it aggravates intestinal injury (113). In mice fed with Western diet, the mortality, systemic inflammation, and bacterial translocation in AP were increased (15). Besides, the diversity of their gut microbiota decreased, the abundance of *E. coli* increased, butyrate producers such as *Lachnoobacter* decreased, and butyrate decreased significantly.

The Mediterranean diet is full of plant food, which provides plentiful dietary fibers, as well as moderate dairy, fish, and olive oil (114). A study found that children with lower Mediterranean diet quality index (KIDMED) were easier to suffer AP (115). Besides, lower KIDMED is related to CP (116). This may be attributed to plentiful dietary fibers and vitamins in the Mediterranean diet. This diet has been shown to be associated with anti-inflammatory effects. The Mediterranean diet can mediate the gut microbiota. More beneficial bacteria, such as *Lactobacilli, Bifidobacteria, Oscillospira*, and *Faecalibacterium*, can be found in a healthier gut microbiota, along with more microbiome-derived beneficial metabolites such as SCFAs (117). The bacteria enhanced by Mediterranean diet can also take part in the metabolism of vitamin, tryptophan, and bile acids (118).

In recent years, soy diet has been found beneficial in CP. Soy bread diet could protect intestinal barrier and reduce inflammation in CP (31). Another study reports that soy diet reduces inflammation and disease severity in CP, which may be due to the reduction in myeloid-derived suppressor cells (MDSC), which reduce reactive oxygen species (ROS) in the pancreas microenvironment (119).

### Prebiotics

Prebiotics refer to the food ingredients that cannot be digested but can be fermented by the gut microbiota in the diet (120). Chitosan oligosaccharides (COS) reduced pancreatic inflammatory infiltration and oxidative stress in SAP mice (121). COS improved the SAP-related microbiota dysbiosis, increased the probiotic *Akkermansia*, and reduced detrimental microbes such as *Escherichia–Shigella* and *Enterococcus*. Galactooligosaccharides (GOS) can significantly increase the number of fecal *Bifidobacteria* and improve intestinal barrier in SAP (122). Besides, fructo-oligosaccharides and polydextrose may protect intestinal barrier function in pancreatitis patients (123).

### Herbal medicine

The active ingredient of some herbal medicine has been demonstrated to improve the gut microbiota by promoting commensal microbes and restraining detrimental microbes (124). The typical herbal medicinal used in pancreatitis included rhubarb anthraquinones, salvia miltiorrhizae, berberine, and resveratrol.

Studies found that rhubarb anthraquinones can protect damaged intestinal barrier in SAP (125). Anthraquinones are found to improve microbiota and increase the abundance of *A. muciniphila* (126). Salvia miltiorrhizae can alleviate multiple organ damage, promoting immune function and. thereby. improving the survival rate of SAP rats (127). Salvia miltiorrhizae has been found to regulate intestinal microbiota via regulating the abundance of bacteria (124). Berberine is able to alleviate pancreatitis via protecting damaged intestinal barrier (128). Berberine increases SCFAs and SCFA producers such as *Blautia*, *Allobaculum, Butyricicoccus*, and *Phascolarctobacterium* (129, 130). Resveratrol can treat AP via anti-inflammatory and antioxidant mechanisms (131). Resveratrol is found to enhance the abundance of *Bifidobacterium* and *Lactobacillus*, and promote the catabolism of BAs (132). Moreover, bacteria may transform some medicines, such as Saikosaponin (133, 134) and Chinese yam polysaccharides (135), into their active ingredient.

### Fecal microbiota transplantation

With the recognition of the pivotal role played by the gut microbiota in disease pathogenesis, researchers commenced exploring the novel approach of fecal microbiota transplantation (FMT) for therapeutic intervention. In the past few years, the value of FMT has been seen in some diseases such as Crohn’s disease and ulcerative colitis (136, 137). With regard to pancreatitis, some progress has been shown recently. FMT showed its ability to alter the microbiota and reverse the detrimental effects of harmful microbes in pancreatitis models (121). Normobiotic FMT was found to mitigate AP-induced gut microbiota dysbiosis and alleviate AP through ameliorating the mitochondrial dysfunction, oxidative stress, and inflammation (16). NMN increased after FMT and stimulated SIRT3 signaling to alleviate AP. In CP, FMT also showed its potential in affecting pancreatic fibrotic progression via immune regulation (138). In short, FMT has a great potential in the treatment of pancreatitis.

## CONCLUSION AND PROSPECT

**Fig 3:**
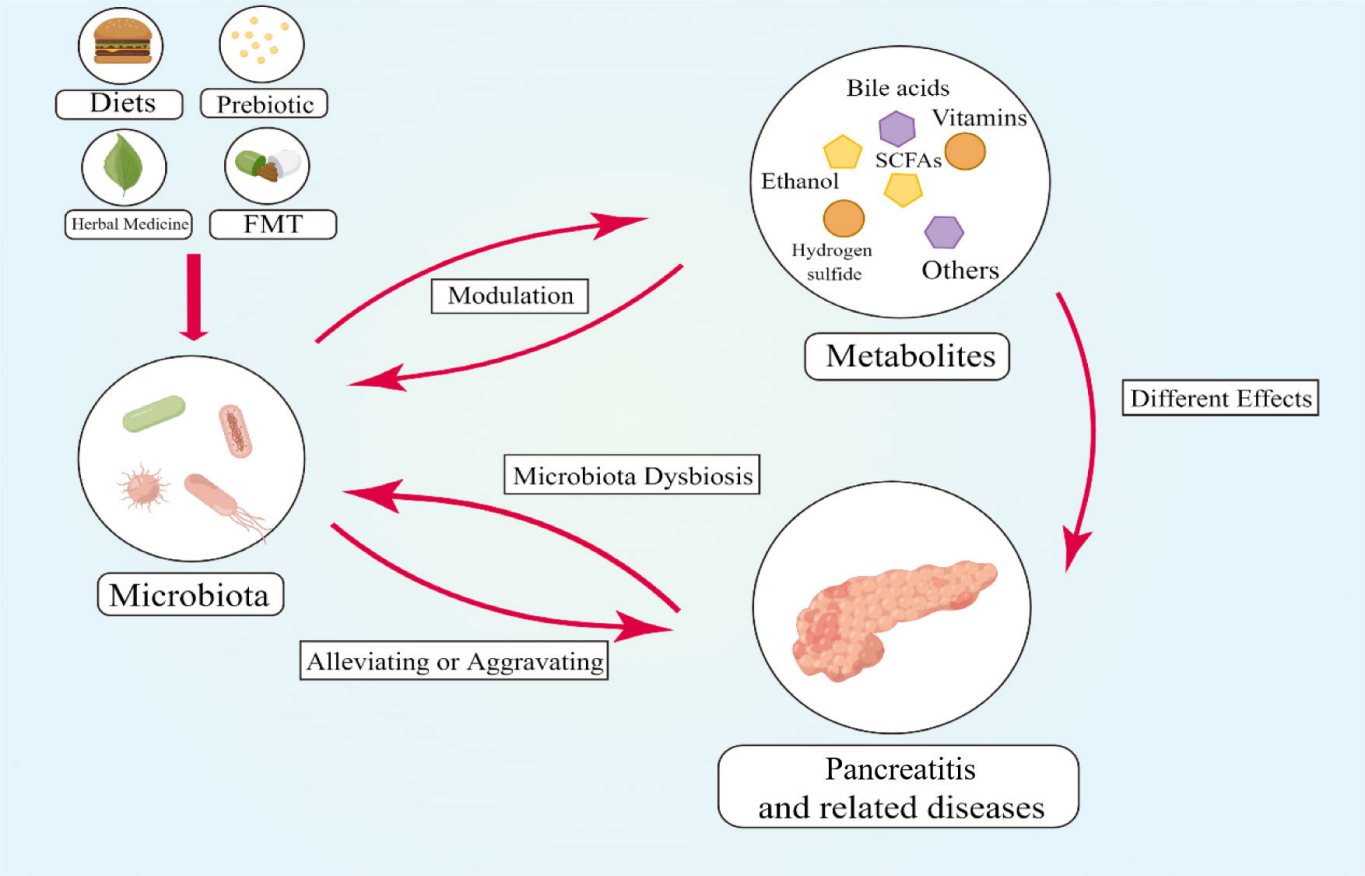
Overview of the relationship between the gut microbiota, metabolites, and pancreatitis as well as the new intervention measures related to the gut microbiota and metabolites. The gut microbiota affects metabolites and the progression of pancreatitis. Metabolites make different effects on the gut microbiota and pancreatitis. Pancreatitis can cause microbiota dysbiosis. Intervention measures, such as diets, prebiotic, herbal medicine, and FMT, can modulate the gut microbiota to alleviate pancreatitis.

Pancreatitis, featuring inflammation, exudation, hemorrhage, necrosis, and fibrosis in the pancreas, may develop into severe acute pancreatitis, chronic pancreatitis, pancreatic cancer, and diabetes mellitus, which lead to lower quality of life and even death. Microbiome and its metabolites play an important part in pancreatitis and its related diseases. The reduction of microbiota diversity and the alteration of metabolite profile have been documented and are associated with pancreatitis. Acute pancreatitis, chronic pancreatitis, autoimmune pancreatitis, and associated pancreatic diseases, respectively, have relationships with different microbiome and metabolites, which may be a method to differentiate these diseases from others. Metabolites, such as SCFAs, BAs, vitamins, H_2_S, alcohol, and so on, have a close relationship with pancreatitis as a critical bridge between pancreatitis and microbiome. Many intervention measures, such as diet, prebiotic, herbal medicine, and FMT, exhibited their vital role as a modulator of gut microbiota and related metabolites (Fig. 3). However, the relationship between pancreatitis and microbial metabolites remains to be studied. In order to better understand the association between pancreatitis and microbiome, future studies might concentrate on how microbiome affects pancreatitis via its metabolites and what other substance may regulate this progression. Moreover, the biological mechanisms, such as fibrosis, inflammation, and cell immune and signal pathways, involved in the effects of microbiome–metabolite interaction on pancreatitis remain to be clarified.

As parts in the progression of pancreatitis, the gut microbiome, metabolites, and their modulation substances are potential targets to alleviate and even treat pancreatitis. For example, microbiome and metabolites may be excellent biomarkers to predict the progression of pancreatitis. FMT technology, based on the principle of regulating the flora to treat disease, has been flourishing in recent years, and as its good efficacy has been proven in many diseases, it may be used as a potential treatment for pancreatitis. Moreover, genetically modified bacteria, which can be applied to alter the component of gut metabolites, provide new technical support for the diversified ecological therapy of AP. However, the effects of treatments based on microbial metabolites are not yet clear. Thus, metabolomics studies, microbiomics studies, and clinical trials are essential to further explore the best therapy for pancreatitis, whether it be beneficial bacteria supplements, beneficial metabolite supplements, proper diet, prebiotics, or herbal medicine.
